# Cytoskeletal changes induced by allosteric modulators of calcium-sensing receptor in esophageal epithelial cells

**DOI:** 10.14814/phy2.12616

**Published:** 2015-11-24

**Authors:** Solange Abdulnour-Nakhoul, Karen L Brown, Edd C Rabon, Youhanna Al-Tawil, Mohammed T Islam, John J Schmieg, Nazih L Nakhoul

**Affiliations:** 1 Medicine/Gastroenterology, Tulane Medical SchoolNew Orleans, Louisiana; 2 South Louisiana Veterans Health Care System (SLVHCS)New Orleans, Louisiana; 3 Medicine/Nephrology, Tulane Medical SchoolNew Orleans, Louisiana; 4 Pediatric Gastroenterology and Nutrition-GI for Kids, East Tennessee Children’s HospitalKnoxville, Tennessee; 5 Pathology, Tulane Medical SchoolNew Orleans, Louisiana

**Keywords:** Adherens junction, calcimimetics, E-cad/CTF2, esophagus, stratified squamous epithelium

## Abstract

The calcium-sensing receptor (CaSR), a G-protein-coupled receptor, plays a role in glandular and fluid secretion in the gastrointestinal tract, and regulates differentiation and proliferation of epithelial cells. We examined the expression of CaSR in normal and pathological conditions of human esophagus and investigated the effect of a CaSR agonist, cinacalcet (CCT), and antagonist, calhex (CHX), on cell growth and cell–cell junctional proteins in primary cultures of porcine stratified squamous esophageal epithelium. We used immunohistochemistry and Western analysis to monitor expression of CaSR and cell–cell adhesion molecules, and MTT assay to monitor cell proliferation in cultured esophageal cells. CCT treatment significantly reduced proliferation, changed the cell shape from polygonal to spindle-like, and caused redistribution of E-cadherin and *β*-catenin from the cell membrane to the cytoplasm. Furthermore, it reduced expression of *β*-catenin by 35% (*P* < 0.02) and increased expression of a proteolysis cleavage fragment of E-cadherin, Ecad/CFT2, by 2.3 folds (*P* < 0.01). On the other hand, CHX treatment enhanced cell proliferation by 27% (*P* < 0.01), increased the expression of p120-catenin by 24% (*P* < 0.04), and of Rho, a GTPase involved in cytoskeleton remodeling, by 18% (*P* < 0.03). In conclusion, CaSR is expressed in normal esophagus as well as in Barrett’s, esophageal adenocarcinoma, squamous cell carcinoma, and eosinophilic esophagitis. Long-term activation of CaSR with CCT disrupted the cadherin–catenin complex, induced cytoskeletal remodeling, actin fiber formation, and redistribution of CaSR to the nuclear area. These changes indicate a significant and complex role of CaSR in epithelial remodeling and barrier function of esophageal cells.

## Introduction

Stratified squamous epithelia play an important role in maintaining a protective barrier between the external and internal environment. This barrier function is dependent on the composition of the cell membranes, the multiplicity of the cell layers, the composition of the intercellular spaces, and the junctions between the cells. Changes in this balance could lead to esophageal diseases including GERD, Barrett’s esophagus, and carcinogenesis (Katz and Kaestner 2002; Marchetti et al. 2003; Goldstein et al. 2007).

Similar to skin keratinocytes, the esophageal squamous cells divide and progress from the basal layers to form the upper differentiated epithelial layers. This ability of cells to proliferate and migrate is fundamental for tissue renewal and homeostasis. Like other epithelial cells, squamous esophageal cells are connected by junctional complexes that limit and regulate paracellular diffusion. This function is essential to preserve the integrity of the barrier and prevent diffusion of acid, allergens, and other noxious material to the basal layers. An increase in the paracellular permeability causes dilated intercellular spaces and is considered a hallmark of reflux disease (Orlando 2008). Alterations in adherens junction proteins are associated with esophageal diseases like eosinophilic esophagitis and cancer. (Stairs Douglas et al. 2011; Abdulnour-Nakhoul et al. 2013)

Calcium-sensing receptor (CaSR) is a G-protein-coupled receptor that was first identified in the parathyroid gland (Brown et al. 1993). It acts as a sensor of changes in extracellular Ca^+2^ and is a mediator of its effects on cellular functions. Ca^+2^ is the primary agonist to activate the receptor, however, other ligands can also activate it including polyvalent cations (e.g., Gd^+3^) that directly activate the receptor (Type I agonists). Other molecules (calcimimetics) including aromatic amino acids, peptides, polyamines metabolites, and aminoglycoside antibiotics are allosteric modifiers (Type II agonists) that activate the receptor, but require the presence of extracellular Ca^+2^. CaSR is becoming an important therapeutic target in kidney disease and hyperparathyroidism as well as a possible target for osteoporosis drugs. Cinacalcet (CCT) is the first calcimimetic approved by the Food and Drug Administration to treat patients suffering from secondary hyperparathyroidism in chronic kidney disease.

CaSR modulates many physiological functions including secretion of peptides, channel activities (Sands et al. 1997), gene expression (Cha et al. 2011), proliferation (Smajilovic et al. 2006; Liu et al. 2009), wound healing (Milara et al. 2010), and cancer (Saidak et al. 2009). In skin keratinocytes, CaSR is reported to affect proliferation, differentiation, and cell–cell junction formation (Tu et al. 2001, 2008; Komuves et al. 2002). In HET1A esophageal cell line, CaSR stimulation activates ERK1 and 2, mobilizes intracellular Ca^+2^, and causes IL-8 and FGF9 secretion (Justinich et al. 2008; Mulder et al. 2009). CaSR is present along the entire small and large intestine (Chattopadhyay et al. 1998; MacLeod 2013b), and has been reported to regulate stomach acid secretion (Busque et al. 2005; Kirchhoff and Geibel 2006). In the large intestine, where it has been studied more extensively, activation of CaSR modulates colonic fluid movement and inhibits cAMP-activated fluid secretion (Geibel et al. 2006). The correlation between CaSR expression and stages of differentiation in human colon tumors (Hizaki et al. 2011) as well as the observation that Ca^+2^ supplements reduced recurrence of colorectal adenomas (Baron et al. 1999) indicate a possible role of CaSR in colon cancer. Although it is prominently expressed in the esophagus, the role of CaSR in this tissue remains poorly defined.

We performed this study to examine the function of CaSR in esophageal epithelial cells. We used the pig esophageal tissue as a model because it has anatomical, physiological, and biochemical features that closely resemble the human esophagus (Christie et al. 1995; Vegesna et al. 2010; Chernichenko et al. 2011). The cephalic part of the pig esophagus has submucosal glands that, similar to the human esophageal glands, secrete bicarbonate and mucus into the lumen (Abdulnour-Nakhoul et al. 2011). The caudal part, about one-third of the esophagus, is devoid of glands and can be used to isolate and culture squamous epithelial cells. These cells unlike immortalized HET-1A cells (Green et al. 2010), can differentiate in culture. Our data indicate that in the human, CaSR is present in normal esophageal tissue as well as in esophageal adenocarcinoma, squamous cell carcinoma, Barrett’s and eosinophilic esophagitis. Using calcimimetics (CaSR agonists) and calcilytics (CaSR antagonists), we identified a major role of the CaSR in regulating proliferation, cytoskeletal architecture, and cell–cell junctional proteins in esophageal epithelial cells.

## Methods

### Human tissues

Archived human samples embedded in paraffin blocks were obtained from the Tulane University Hospital and were used for immunohistochemistry (IHC). Biopsies of normal, reflux esophagitis or eosinophilic esophagitis patients were used for IHC or western blotting. The biopsies were obtained from patients undergoing routine endoscopy procedures after obtaining written consent. The Institutional Review Board of East Tennessee Children Hospital and Tulane University approved the study and all related protocols.

### Primary tissue culture of squamous epithelial cells

Esophagi were obtained from pigs killed in unrelated IACUC-approved studies at the Tulane Medical School or from a local slaughterhouse immediately after animal sacrifice. Tissues were transferred to the lab in an ice-cold HEPES-Ringer solution (containing in mmol/L; Na^+^, 140; Cl^-^, 132.3; K^+^, 5.1; Hepes, 25; Ca^2+^, 1.2; Mg^2+^, 1.2; HPO_4_^2−^, 2.4; H_2_PO_4_^−^, 0.4). The muscularis mucosa was removed, and the mucosal layer was collected and washed several times in sterile cold Dulbecco’s modified Eagle Medium (DMEM) supplemented with: Na^+^-pyruvate (1 mmol/L), fetal bovine serum (2%), HEPES (20 mmol/L, pH 7.4), gentamycin (100 *μ*g/mL), penicillin (100 U/mL), streptomycin (100 *μ*g/mL), and Fungizone (amphotericin B, 2.5 *μ*g/mL). The caudal part of the tissue which is devoid of esophageal glands (Fig.2B) was then minced using two blades, and tissue fragments were washed twice in a plating medium consisting of: Ham’s F12 (47%), DMEM (47%), Serum Replacement (2%), pituitary extract, bovine (10 *μ*g/mL), hydrocortisone (0.36 *μ*g/mL), insulin (10 *μ*g/mL), transferrin (5 *μ*g/mL), endothelial cells growth supplement (7.5 *μ*g/mL), retinoic acid (32 ng/mL), choleratoxin (20 ng/mL), EGF (20 ng/mL), and antibiotics as described above. The minced tissue was resuspended in a plating medium and plated onto cover slips coated with human fibronectin. The cells were incubated at 37°C in 5% CO_2_/95% air. After 6 days, the minced tissue pieces were washed away and the primary culture of squamous cells (stuck to the cover slips) was maintained in similar growth media devoid of antibiotics. Upon confluence, the squamous cells were dispersed with trypsin-EDTA and subcultured up to six times. The final concentration of Ca^2+^ in the control culture media (normally 1.2 mmol/L) was modified according to the experiment. Cinacalcet (CCT) or calhex (CHX) were dissolved in methanol (MetOH) and added to the culture media. The concentration of CCT and CHX were chosen based on previous studies in the literature (Nemeth et al. 2004; Brennan et al. 2015) The concentration of MetOH never exceeded 0.05%. An equal concentration of MetOH was added to the control media.

### MTT cell proliferation assay

Primary cultures of stratified squamous epithelial cells (SSE) obtained as described above were plated at a density of approximately 12,000 cells/cm^2^ in four 24-well plates and were allowed to grow until ∼60% confluent. The cells from each plate were divided into four different treatment groups: control Ca^2+^ (1.2 mmol/L); low Ca^2+^ (0.4 mmol/L); CCT (2.5 μmol/L) in 0.4 mmol/L Ca^2+^; and CHX (5 μmol/L) in control media. Proliferation was measured at 24 h for the first plate, 48 h, 72 h, and 96 h consecutively for the other three plates. Following treatment, 3-(4,5-dimethylthiazol-2-yl)-2,5-diphenyltetrazolium bromide, MTT, (Invitrogen, CA) was added to each well. The dye is reduced to purple in the mitochondria of live cells. The cells were incubated at 37°C for another 4 h, the medium was removed, and SDS-HCl solution was added to each well to solubilize the purple formazan product. Absorbance was then read at a wavelength of 570 nm using a FLUOstar Optima plate reader.

### Immunohistochemistry

Tissue sections (5*μ*) were mounted on gelatin-coated slides and immunostained using standard procedures. Paraffin sections were dewaxed in xylene, rehydrated in graded alcohol, and treated with 0.3% H_2_O_2_/methanol to block endogenous peroxidase activity. Sections were blocked and incubated with the primary antibody, washed and incubated with biotinylated secondary antibody against the IgG of the species providing the primary antibody. Sections were then incubated with avidin-biotinylated enzyme complex (ABC, Vector Laboratories, Burlingname, CA). Peroxidase activity was detected using diaminobenzidine (DAB, Sigma, St Louis, MI) as a substrate. Specimens were counterstained with hematoxylin, cleared and mounted in Permount.

Cultured cells on glass coverslips were fixed briefly in 4% methanol-free formaldehyde or cold acetone and stained as described above. For F-actin staining, frozen sections or cultured cells were fixed in 4% methanol-free formaldehyde, washed with PBS, permeabilized in 0.1% Triton, and incubated with Alexa Fluor® 568 phalloidin (Invitrogen) diluted in 1% bovine serum albumin in PBS for 30 min. The sections were counterstained with 4′, 6-diamino-2-phenylindole dihydrochloride (DAPI), a nuclear marker, and mounted in Prolong (Invitrogen). For negative controls, primary antibodies were reacted with their respective fusion protein prior to staining or, alternatively, sections were incubated without the primary antibody. Micrographs were obtained using a Nikon Eclipse 80i microscope and a Spot RT digital camera.

### Western analysis

For protein extraction, cells were lysed in Cell-Lytic (Sigma) in the presence of protease inhibitors. Protein content was quantified using the Pierce BCA protein assay and normalized to 1 mg/mL. The same amount of protein was loaded in each lane of an individual gel. In all experiments, less than 10 *μ*g of protein was loaded per lane, an amount in the optimal linear range of the detection system. Proteins were separated using 10% sodium dodecyl sulfate–polyacrylamide gel electrophoresis (SDS-PAGE) under reducing conditions (Laemmli 1970). Prestained molecular weight markers (LiCor, Lincoln, Nebraska) were run in parallel lanes. Samples were run in duplicates or triplicates on each gel. After electrophoresis, proteins were transferred to a nitrocellulose membrane. The membranes were blocked for nonspecific binding, incubated overnight at 4°C with primary antibodies (see Table1 for concentrations), washed, and incubated with the appropriate secondary antibody conjugated to a fluorescent dye (IRDye 800 CW conjugated goat anti-mouse or IRDye 680 conjugated goat anti-rabbit) at a concentration of 1:7000 (Li-Cor). The immunoreactive complex was visualized using Li-Cor Odyssey Infrared System and analyzed by resident software. The membranes were stripped up to three times using Li-Cor Stripping buffer as directed by the manufacturer, blocked and reprobed as described above.

**Table 1:** Primary antibodies used for immunohistochemistry and Western Analysis

Host-Antibody	Immunogen-clone, source	Protein MW (kD)	Concentrations used
*β*-catenin, mouse monoclonal	Synthetic peptide corresponding to residues 27–37 of human *β*-catenin (Millipore)	∼97	2 *μ*g/mL (WB & IHC)
CaSR, mouse monoclonal	Amino acid peptide sequence 15–29 at the extracellular N-terminus of human CaSR (Sigma-Aldrich)	∼130	1–2 *μ*g/mL (WB) 2–4 *μ*g/mL (IHC)
CaSR rabbit polyclonal	20 amino acid peptide sequence near the C-terminus of human CaSR(Millipore)	∼130	2 *μ*g/mL (WB)
E-Cadherin, Rabbit monoclonal	Synthetic peptide surrounding residue 780 of human E-Cadherin cytoplasmic region (Cell Signaling)	∼130	∼0.1 *μ*g/mL (WB) 0.2–0.4 *μ*g/mL (IHC)
p-120 catenin, mouse monoclonal	C-terminus (Millipore)	90–120	∼1 *μ*g/mL (WB)
Rho (-A, -B, -C) clone 55, Mouse monoclonal	Recombinant human RhoA containing amino-acids 1-155. (Millipore)	22	2 *μ*g/mL (WB)
*β*-actin rabbit monoclonal	synthetic peptide corresponding to the N-terminus of human *β* actin (Licor)	37	0.2 *μ*g/mL (WB
*β*-actin mouse monoclonal	synthetic peptide corresponding to the N-terminus of human *β* actin (Licor)	37	0.2 *μ*g/mL (WB)

*β*-actin was used as a loading control and for normalization. For each sample, duplicates or triplicates from every experiment were pooled and their mean was calculated to yield one data point for that sample. For every experiment, the ratio of protein of interest to *β*-actin in control Ca^+2^ (1.2 mmol/L) was set at one. The reading in each experimental condition was normalized to its respective *β*-actin reading and compared to one. Statistical analysis was performed using two-tailed Student’s *t*-test. The primary antibodies used for WB and IHC are listed in Table1.

### Total RNA isolation, reverse transcription, and amplification of mRNA

Total RNA was isolated from homogenized tissues or cultured cells collected in Trizol, according to manufacturer’s instructions. Primers were obtained from Integrated DNA Technologies (Coralville, IA). The forward and reverse primers for porcine CaSR were, respectively, 5′-CCTGGACTGAGCCTTTTGGGATTG-3′ and 5′-CTGGAGCGCTGGCGGGAGACG-3′. Reverse transcription and amplification of target sequences was performed using SuperScript III One-Step RT-PCR System for end point detection (Invitrogen, Carlsbad, CA) according to manufacturer’s instruction. RT-PCR products were resolved by electrophoresis using a 2.5% agarose gel containing 10 *μ*g/mL ethidium bromide. The approximate size of each product was determined by comparison to a DNA ladder (Invitrogen). PCR products were purified and sequenced using Applied Biosystems 3130xl Genetic Analyzer to confirm gene identity (Tulane Medical Center Core Facility).

### Apoptosis and viability assays

Cells were cultured in 25 cm^2^ flasks and were allowed to reach ∼60% confluence. Cells were then divided into groups, the first one was nontreated control switched to low Ca^+2^ media (0.4 mmol/L). Two groups were treated with CCT/0.4 mmol/L Ca^+2^ (2.5 μmol/L) for 24 h or 48 h. One group of cells was treated with H_2_O_2_ for 4 h at the same time CCT treatment was initiated, then was switched to control media for the rest of 24 h and used as a positive control for apoptosis. At the specific time frame, the cells were harvested with 0.05% trypsin, collected in media, and stained for Annexin V and propidium iodide (PI) using the FITC AnnexinV Apoptosis Detection Kit (BD Biosciences, San Jose, CA) and analyzed by flow cytometry to determine the percentage of apoptotic and necrotic cells. Annexin V^−^/PI^−^ were defined as live cells Annexin V^+^/PI^−^ cells as apoptotic, and Annexin V^+^/PI^+^ cells as necrotic.

For cell viability assay, 0.2% trypan blue was added to the culture and incubated for 2 min. The dye was removed and a little media was added to the coverslip to improve visualization. The cells showing uptake of the dye were counted as dead. Viability was measured in three fields (% alive/total number of cells).

### Chemicals

Chemicals were obtained from Sigma. Cinacalcet hydrochloride and calhex were obtained from Toronto Research Chemicals (TRC, Toronto, CA) and were dissolved in methanol before they were added to the cultures. The concentration of methanol never exceeded 0.05%.

### Statistical analysis

Data are presented as mean ± SEM. Data were analyzed using two sample Student’s *t*-test unless otherwise indicated. *N* is the number of experiments.

## Results

### Immunolocalization of the CaSR in the esophagus

**Figure 1:**
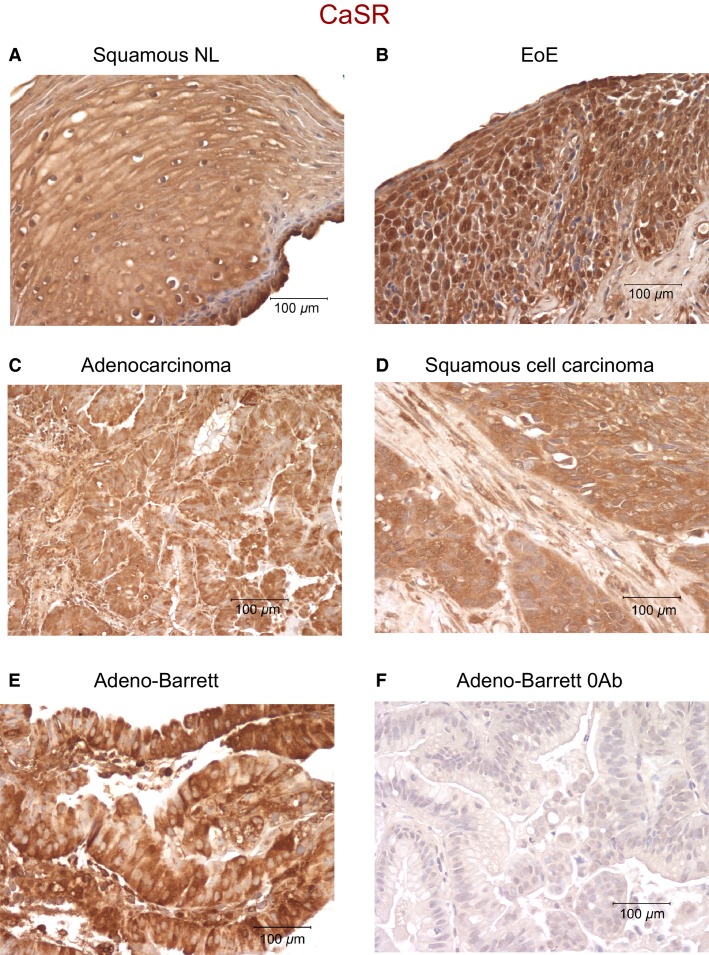
CaSR expression in human esophageal tissues. Positive staining for CaSR is indicated by brown deposits. (A) shows a section from a normal (NL) esophageal biopsy, (B) biopsy from eosinophilic esophagitis patient (EoE), (C) adenocarcinoma, (D) squamous cell carcinoma, (E) Barrett’s adenocarcinoma. All sections were positive for CaSR indicating the presence of the receptor in the esophageal tissues. (F) shows an esophageal section where the primary antibody was omitted from the staining procedure. The experiment was repeated three times using samples from two adenocarcinoma patients, two adenocarcinoma with Barrett’s patients, three squamous cell carcinoma patients, three eosinophilic esophagitis, and three normal patients.

Normal human esophageal squamous epithelium immunostained for CaSR showed positive staining (brown, Fig.1A). Esophageal tissues from patients diagnosed with eosinophilic esophagitis (Fig.1B), adenocarcinoma (Fig.1C), squamous cell carcinoma (Fig.1D), or Barrett’s esophagus (Fig.1E), all showed strong positive staining for CaSR. Figure1F is a negative control where the primary antibody was omitted from the staining procedure. This experiment indicates that the receptor is present in normal tissue as well as in a number of pathological conditions of the esophagus.

**Figure 2:**
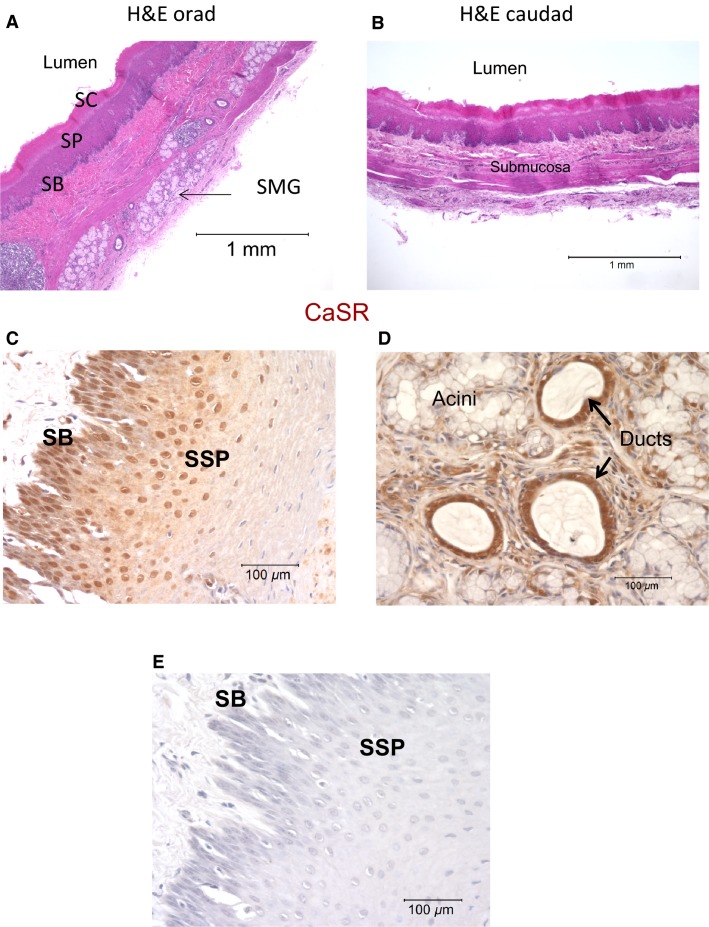
Porcine esophageal histology and CaSR expression by immunohistochemistry (A) Cross section from orad region of the pig esophagus stained with H&E showing submucosal glands (SMG); SB (stratum basalis), SSP (stratum spinosum), SC (stratum corneum). (B), Cross section from caudad region devoid of glands. (C), Immunostaining of CaSR (brown deposits) in the stratified squamous epithelium. CaSR is distributed in the basal and suprabasal layers. (D), shows staining for CaSR in submucosal glands and strong expression in the ducts. The experiment was repeated three times using tissues from three different pigs. (E) shows a tissue section where the primary antibody was omitted from the staining procedure.

For this study, we immunolocalized CaSR in the pig esophagus. The pig esophagus similar to the human bears submucosal glands. As shown in Figure2A, a cross section of the orad area of pig esophagus stained with hematoxylin–eosin shows submucosal glands (SMG), while Figure2B shows a section of the caudal area that is devoid of SMG. Immunostaining for CaSR (brown deposits) showed the distribution of the receptor in stratified squamous epithelium (Fig.2C). The intensity of staining for CaSR was strongest in the basal and suprabasal layers. Figure2D shows an area of esophageal epithelium bearing submucosal glands with immunostaining for CaSR, where the intensity of staining was strongest in the glandular ducts. Figure2E is a negative control where the primary antibody was omitted from the staining procedure.

### Tissue culture of the squamous epithelium

**Figure 3:**
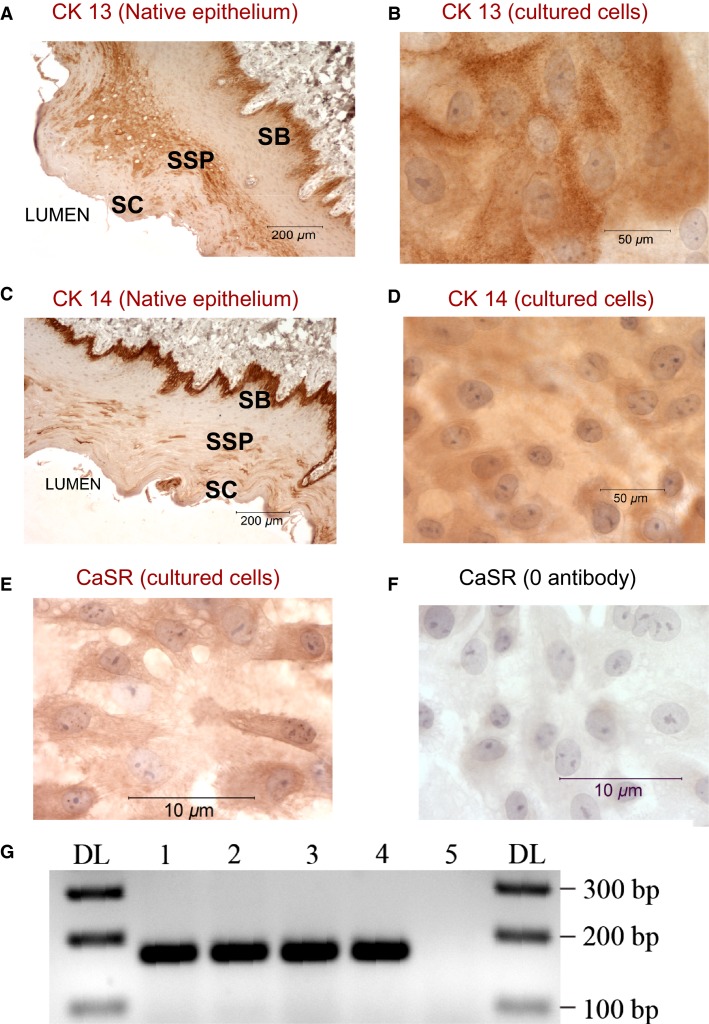
Characterization of the cells in culture. CK13 and CK 14 staining of esophageal section (A and C respectively) and of cultured squamous cells (B and D). Brown deposits indicate positive staining in basal and suprabasal layers of the epithelium. Cells in culture also stained positive for CK13 and Ck14 indicating their similarity to epithelial cells of the esophagus. Representative data from 3 different experiments. SB (stratum basalis), SSP (stratum spinosum), SC (stratum corneum). E) CaSR expression in cultured esophageal cells by IHC. (E), shows immunostaining of SSE cells grown in control 1.2 mmol/L Ca^+2^. SSE cells stained positive for CaSR (brown deposits). (F), negative control, the primary antibody to CaSR was omitted from immunostaining procedure. (G), RT-PCR amplification of CaSR products performed on extracted total RNA: lane 1, cultured cells from esophageal submucosal glands (SMG); lane 2, cultured cells from squamous epithelium, lane 3, native squamous esophageal tissue; lane 4, esophageal submucosal glands (SMG). Lane 5 is a negative control where all the reactants (as in lane 3) are present but *Taq* polymerase was omitted from the reaction. The first and last lanes are DNA ladder. A prominent band at the expected size of 170 bp confirmed the expression of CaSR in squamous esophageal tissue, SMG, and in cultures derived from these tissues. The sequences of the products are shown in Table2. Representative data are from four different experiments.

To characterize the role of CaSR in the esophagus, we established a primary culture of squamous epithelial cells from the caudal part (devoid of glands) of pig esophagus as described in Methods section. After few days, the cultured squamous epithelial cells (SSE) formed a sheet of cells with a cobblestone appearance. To confirm the epithelial origin of these cells, we stained them for cytokeratins (CK), which are cytoskeletal intermediate filament proteins expressed preferentially in tissues of epithelial nature (Moll et al. 1982; Boch et al. 1997). Figure3A shows CK13 staining in sections of native tissue and Figure3B shows that the primary cultures stained positive for CK13 indicating their similarity to the basal and suprabasal epithelial cells of the native esophagus tissue. Staining with CK 14 further confirmed their epithelial origin and is shown in Figure3C for native tissue and 3D for cultures.

We validated the presence of CaSR in the cultured esophageal cells using immunostaining and RT-PCR. Figure3E shows SSE cells stained for CaSR where positive staining is indicated by brown deposits, while Figure3F shows cells stained simultaneously, but the CaSR antibody was omitted from the staining procedure (negative control).

To confirm the presence of mRNA encoding the CaSR, RT-PCR experiments were performed (Fig.3G) using total RNA extracted from native squamous esophageal tissue (lane 3), native submucosal esophageal glands (lane 4) (collected as described in details in [Abdulnour-Nakhoul et al. 2007]), or cultured cells derived from these tissues (respectively lanes 2 and 1). The experiment showed a clear band at the expected size of 170 bp confirming the expression of the receptor in these tissues and cells. Lane 5 is a negative control where the *Taq* polymerase was omitted from the reaction. The PCR products were purified and sequenced. Nucleotide sequences from all tissues were blasted against the porcine CaSR sequence and found to be similar to that sequence at >98%. The nucleotide sequences are listed Table2

**Table 2:** Nucleotide sequences of the purified PCR products from esophageal native tissues and derived cultures. Sequences from all tissues were blasted against the porcine CaSR sequence and found to be similar to that sequence at >98%

Stratified squamous epithelium native	AAYTATKCGKCTGGGCATTTTCCTCACCGCCTTTGTGCTGGGCGTCTTCATCAAGTTCCGAAACACGCCCATCGTCAAGGCCACCAACCGGGAGCTCTCCTACCTTCTCCTCTTCTCCCTGCTCWGCTGCTTCTCCAA (Forward) ARAWGCAARCTCGGTTGGTGGCCTTGACGATGGGCGTGTTTCGGAACTTGATGAAGACGCCCAGCACAAAGGCGGTGAGGAAAATGCCCAGCACGGCAAAGAGGGTGAGTGCAATCCCAAAAGGMTMAGTCCAGGA (Reverse)
Stratified squamous cultured cells	AMYTATGCGKMTGGGCATTTTCCTCACCGCCTTTGTGCTGGGCGTCTTCAWCAAGTTCCGAAACACGCCCATCGTCAAGGCCACCAACCGGGAGCTCTCCTACCTTCTCCTCTTCTCCCTGCTCWGCTGCTTCTCCAA (Forward) AAWGGGARCTCGGTTGGTGGCCTTGACGATGGGCGTGTTTCGGAACTTGATGAAGACGCCCAGCACAAAGGCGGTGAGGAAAATGCCCAGCACGGCAAAGAGGGTGAGTGCAATCCCAAAAGGMTCAGTCCAGGA (Reverse)
Submucosal glands native	CCCYCTTKCGKCTGGGCATTTTCCTCACCGCCTTTGTGCTGGGCGTCTTCATCAAGTTCCGAAACACGCCCATCGTCAAGGCCACCAAYCGGGAGCTCTCCTACCTTCTCCTCTTCTCCCTGCTCWGCTGCTTCTCC (Forward) ARTACGARCTCCGRTTGGTGGCCTTGACGATGGGCGTGTTTCGGAACTTGATGAAGACGCCCAGCACAAAGGCGGTGAGGAAAATGCCCAGCACGGCAAAGAGGGTGAGTGCAATCCCAAAAGGMTCAGTCCAGGA (Reverse)
Submucosal glands cultured cells	CCCYYATTKCGKCTGGGCATTTTCCTCACCGCCTTTGTGCTGGGCGTCTTCATCAAGTTCCGAAACACGCCCATCGTCAAGGCCACCAACCGGGAGCTCTCCTACCTTCTCCTCTTCTCCCTGCTCTGCTGCTTCTCCAA (Forward) ARCAGRARCTCGGTTGGTGGCCTTGACGATGGGCGTGTTTCGGAACTTGATGAAGACGCCCAGCACAAAGGCGGTGAGGAAAATGCCCAGCACGGCAAAGAGGGTGAGTGCAATCCCAAAAGGMTCAGTCCAGGA (Reverse)

### Chronic effect of low Ca^2+^, calcimimetics, and calcilytics on cell morphology

**Figure 4:**
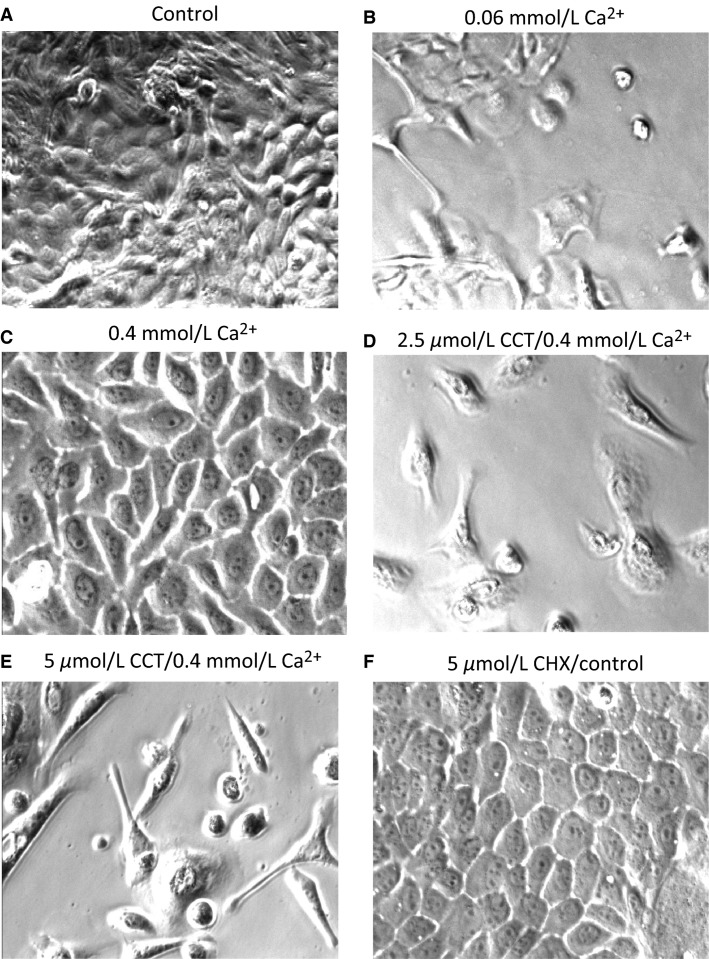
Effect of cinacalcet (CCT) and calhex (CHX) treatment on cultured squamous cells morphology. (A), the cells were maintained in normal pig media (1.2 mmol/L Ca^+2^) while in (B) they were maintained in media containing 0.06 mmol/L Ca^+2^. (C), cells maintained in 0.4 mmol/L Ca^+2^. (D), cells were treated for 24 h with 2.5 μmol/L CCT/0.4 mmol/L Ca^+2^. In (E), the cells were treated for 24 h with 5 μmol/L CCT/0.4 mmol/L Ca^+2^. The cells that were exposed to CCT lost their polygonal cobblestone appearance and became spindle shaped a transformation more pronounced at a higher concentration of CCT. (F), the cells were treated for 24 h with CHX and did not appear different from control (5 μmol/L). Representative data from five different experiments.

To investigate the effect of Ca^2+^ on the growth of cultured esophageal squamous cells, we plated the cells, first in normal Ca^2+^ (1.2 mmol/L) to allow them to attach for 2 h, after which we switched the media to concentrations of Ca^2+^ of 0.06 mmol/L, 0.1 mmol/L, or 0.4 mmol/L, and the cells were left to grow for few days. We also wanted to establish the lowest concentrations of Ca^+2^ at which proliferation remained optimal. Cells grown in normal media containing 1.2 mmol/L Ca^+2^ grew robustly and formed a confluent sheet of cells with typical cobblestone appearance (Fig.4A). On the other hand, cells grown in 0.06 mmol/L Ca^+2^ did not proliferate at all (Fig.4B). Similarly, cells plated in 0.1 mmol/L Ca^2+^ showed sparse growth and arrested mitosis. However, when cells were plated at a concentration of 0.4 mmol/L Ca^+2^, they proliferated normally, had normal cobblestone appearance and reached confluence at a time frame similar to the cells grown in media at control Ca^2+^ of 1.2 mmol/L (Fig.4C).

To check the effect of CaSR activation by calcimimetics on cell growth and proliferation, we performed a set of experiments in which equal numbers of primary SSE cells were plated in 24-well plates. The cells were left to grow for 48 h or until they reached ∼60% confluence in normal culture media (1.2 mmol/L Ca^2+^). The cells were then divided into groups of 4 wells each as follows: (1) Control media; (2) 0.4 mmol/L Ca^+2^; (3) 2 μmol/L CCT/0.4 mmol/L Ca^+2^; and (4) 5 μmol/L CCT/0.4 mmol/L Ca^+2^. We used this lower concentration of Ca^+2^ with CCT because at normal Ca^+2^ concentrations, CaSR may be fully activated by Ca^+2^, its main agonist, and might not respond further to CCT (Nemeth et al. 2004). The cells grown in 0.4 mmol/L had normal growth and appearance as shown in Figure4C. However, the cells that were exposed to CCT for 24 h, lost their polygonal cobblestone appearance and became spindle shaped (Fig.4D). This transformation was more pronounced at a higher concentration of CCT as shown in Figure4E. Simultaneously, we investigated the effect of negative allosteric modulation of CaSR on the cells. The cells were grown as described above and treated with 5 μmol/L Calhex (CHX) for 48 h. The cells treated with CHX had a normal appearance and reached confluence at a time frame similar to control untreated cells (Fig.4F). These data indicated that CCT had a significant effect on the shape and proliferation of the squamous cells in culture.

### Viability of cells treated with CCT or CHX

Because of the prominent changes in cell morphology in cells treated with CCT, we needed to confirm their viability. To do so, we used two techniques, trypan blue uptake or FITC-Annexin V staining and flow cytometry analysis. Trypan blue uptake in cells treated with 2.5 or 5 μmol/L CCT/0.4 mmol/L Ca^+2^ for up to 72 h was not different from control (data not shown).

To use FITC-Annexin V staining, cells were plated and grown in 25 cm^2^ flasks for 24 h. Cells were divided into groups, the first was a control nontreated group grown in low Ca^+2^ (0.4 mmol/L). The other groups were treated with CCT/0.4 mmol/L Ca^+2^ (2.5 or 5 μmol/L) for 24 h, 48 h, or 72 h. One group of cells was treated with H_2_O_2_ for 4 h at the same time CCT treatment was initiated, then was switched to control media for the rest of 24 h and used as a positive control for apoptosis. Flow cytometry was performed on all groups simultaneously as described in Methods. Our data indicated that, at every time period, there was no significant difference in the number of live cells between control and CCT-treated cells (Table3). These results were opposite to the results of treatment with H_2_O_2_ (positive control) where there was a large increase in the number of apoptotic cells and an evident reduction in number of live cells (Table3).

**Table 3:** Experiments showing the number of viable, early apoptotic, late apoptotic, and necrotic cells in nontreated cells (0.4 mmol/L Ca^+2^) and cells treated with CCT/0.4 mmol/L Ca^+2^. Cells were stained for Annexin V and propidium iodide (PI) and analyzed by flow cytometry to determine the percentage of apoptotic and necrotic cells. Annexin V^−^/PI^−^ were defined as live cells, Annexin V^+^/PI^−^ cells as apoptotic, and Annexin V^+^/PI^+^ cells as necrotic. The experiments were repeated three times. The experiment with hydrogen peroxide is a positive control

	% of total cells			
	Q3	Q4	Q2	Q1
	Live cells	Early apoptosis	Late apoptosis	Necrosis
0.4 mmol/L Ca^+2^-24 h	93 ± 2	3 ± 1	3 ± 1	2 ± 1
CCT-2.5 μmol/L-24 h	96 ± 1	2 ± 1	2 ± 2	1 ± 1
0.4 mmol/L Ca^+2^-48 h	89 ± 1	3 ± 1	6 ± 1	2 ± 1
CCT-2.5 μmol/L-48 h	84 ± 1	4 ± 1	11 ± 2	1 ± 1
H_2_O_2_ (0.5 mmol/L) 4 h positive control	7	70	23	1

### Effect of CCT and CHX on cell proliferation

**Figure 5:**
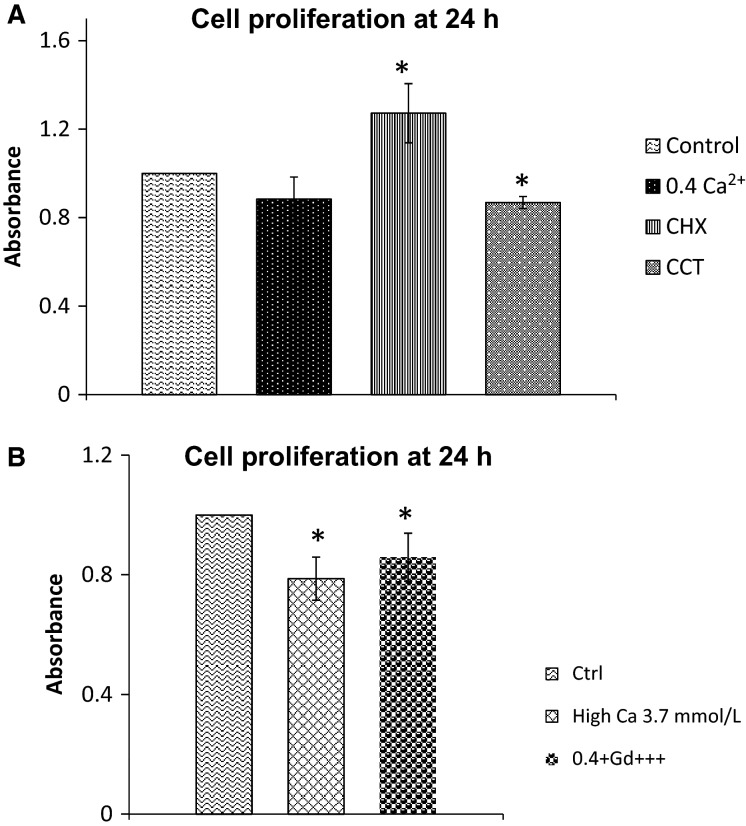
Effect of CaSR modulators on proliferation of squamous cells in culture. Cell proliferation was measured by MTT assay.(A), bar graphs showing proliferation in different treatment groups at 24 h. In CCT, the rate of proliferation was 87 ± 3% of control (*P* < 0.001), whereas the rate of proliferation in CHX-treated cells was 127 ± 13% (*P* < 0.007). Data are from four different experiments. (B), bar graphs showing cell proliferation at 24 h in media containing high Ca^+2^ (3.7 mmol/L) or Gd^+3^. In high Ca^+2^, the rate of proliferation was 78 ± 7% compared to control (*P* < 0.01). In 0.4 mmol/L Ca^+2^/Gd^+3^, cell proliferation was 86 ± 8% compared to control (*P* < 0.04). * indicates significant difference from control. Data are from five different experiments.

To monitor proliferation of esophageal cells treated with calcimimetics or calcylitic, we used MTT assay as detailed in Methods. The dye intensity, measured by spectrophotometry after solubilizing the formazan product is proportional to the number of live cells in the cultures. We compared the dye intensity in the treated cells to the dye intensity in control cells. The cells were plated in 24-well plates and monitored over the course of 96 h as described in Methods section. For each group of cells (Control, 0.4 mmol/L Ca^+2^, CCT/0.4 mmol/L Ca^+2^ or CHX), the absorbance readings at 48 h, 72 h, and 96 h were normalized to the control reading at 24 h for each group. CCT caused a significant decrease in proliferation at 24 h and 72 h (by 13% and 37%, respectively, as compared to control, *P* < 0.003), whereas CHX caused a significant increase in proliferation at 24 h (by 27%, *P* < 0.007) Figure5A shows the data from this experiment at 24 h.

To confirm the CaSR-mediated effect on proliferation, we tested the effect of type 1 CaSR agonists, we monitored cell proliferation at 24 h after activation of CaSR with high Ca^+2^ in the tissue culture media (3.7 mmol/L) or by addition of gadolinium (Gd^3+^, 100 μmol/L) a multivalent cation and a potent Type I agonist of CaSR. High Ca^+2^ and Gd^3+^, both reduced proliferation significantly (by 22% and 14%, respectively, *P* < 0.04) compared to control (Fig.5B).

### Chronic effects of CCT and CHX on CaSR distribution and expression

**Figure 6:**
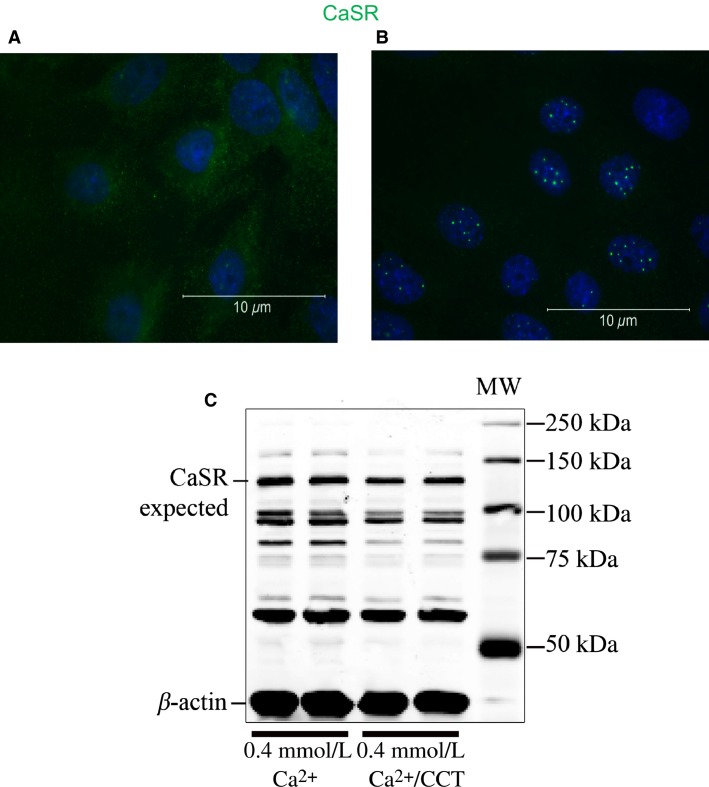
Effect of cinacalcet treatment on CaSR expression. (A) shows staining for CaSR (green) in cells maintained in 0.4 mmol/L Ca^+2^. (B), in cells treated for 48 h with 2.5 μmol/L CCT/0.4 mmol/L Ca^+2^ CaSR was localized to an area very close to the nucleus. (C) Western blot analysis of protein extracts from nontreated cells in 0.4 mmol/L Ca^+2^ and cells treated with CCT/0.4 mmol/L Ca^+2^ for 48 h. The blot was incubated with antibodies to CaSR and *β*-actin. CaSR was present at the expected MW of 130 kD in extracts from nontreated and CCT-treated cells. Other reactive bands were also present at ∼90, 80, and 60 kD possibly resulting from proteolytic degradation of the receptor. Representative data are from three different experiments.

Allosteric modulation of CaSR has been reported to stabilize the receptor and increase its expression at the cell membrane (Cavanaugh et al. 2012). On the other hand, sustained activation of GPCRs could be followed by desensitization or internalization of the receptor (Lefkowitz and Shenoy 2005). We therefore investigated if the cytoskeletal changes observed with CCT treatment are due to changes in expression of CaSR in esophageal cells. To do this, we studied the distribution of CaSR by IHC after 48 h treatment with CCT. Under control conditions (Fig.6A), CaSR staining was diffuse in the cell membrane and cytoplasm. Few cells showed perinuclear distribution of the receptor. Treatment with CCT (2.5 μmol/L) for 48 h caused redistribution of the receptor to the perinuclear area and the number of cells showing this redistribution was increased (Fig.6B).

**Figure 7:**
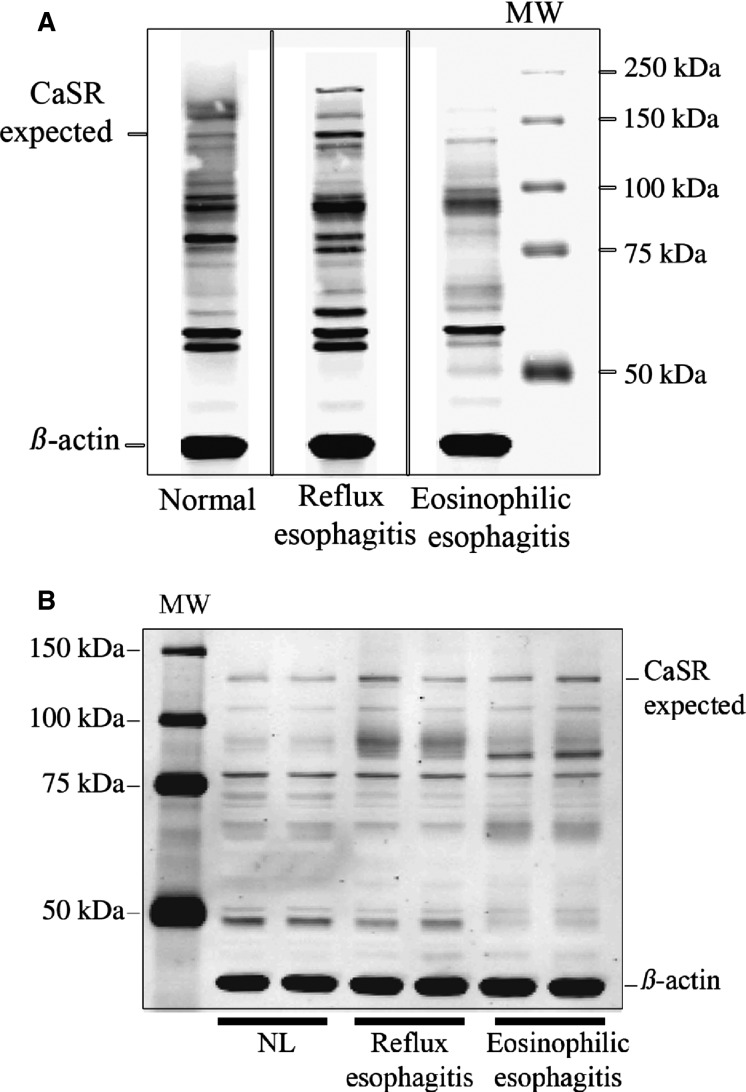
Western analysis of lysates from human biopsies. (A) Immunoblot showing the distribution of CaSR in biopsies from normal (NL) esophagus, reflux esophagitis (RE), and eosinophilic esophagitis (EoE) tissues. The blot was stained with a mouse antibody (Sigma-Aldrich) raised against human CaSR (amino acid peptide sequence 15–29 at the extracellular N-terminus of human CaSR). A CaSR band was evident at the expected MW of 130 kD and at 160 kD (the glycosylated form). However, similar to the blot from pig squamous cells other reactive bands were also present at ∼90, 80, and 60 kD possibly resulting from proteolytic degradation of the receptor. Vertical solid lines indicate noncontiguous lanes of the same gel. *B*: Immunoblot from a different set of biopsies; normal, RE and EoE patients. The blot was stained this time with a polyclonal antibody to CaSR raised against the C-terminus of human CaSR (Millipore). The blot shows a band at the expected MW of 130 kD, but it also shows clusters of bands at smaller MWs.

We then used Western analysis to determine the effect of CCT on CaSR expression. Figure6C shows no significant difference between the expression level of CaSR protein at 130 kD in cells grown in 0.4 mmol/L Ca^+2^ and cells treated with CCT/0.4 mmol/L Ca^+2^(values normalized to *β*-actin). It is important to note that the immunoblot showed the expected CaSR band at 130 kD but also showed other bands at ∼90, 70, and 60 kD probably indicating immature or degraded receptors (Grant et al. 2011; Breitwieser 2013). The antibody we used for this experiment was against amino acid peptide sequence 15–29 at the extracellular N-terminus of human CaSR (Sigma-Aldrich, St Louis, MO). We observed a similar pattern of staining (multiple bands) when we repeated the experiment using protein extracts from human esophageal biopsies from normal, reflux disease or eosinophilic esophagitis patients or if we used antibodies against other peptide sequences of N or C-termini of the CaSR, confirming that the presence of multiple bands was not an artifact (Fig.7A and B). It is of interest to note that the pattern of staining for CaSR was different in different pathological conditions of the human esophagus.

The presence of multiple bands at MW <108 kD was also reported by other studies on *wild-type* and mutant isoforms of CaSR expressed in HEK cells, (White et al. 2009) and was attributed to proteosomal degradation of the receptor.

### Effect of CCT on junction proteins

**Figure 8:**
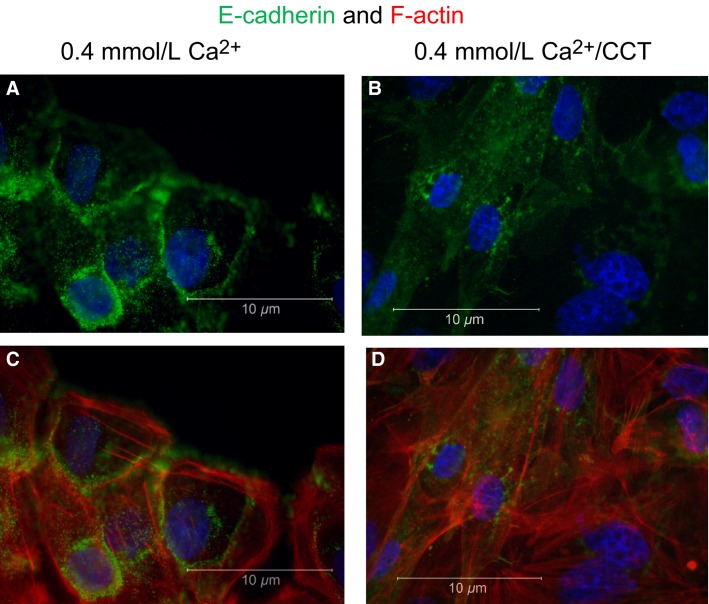
Effect of cinacalcet on E-cadherin distribution. The figure shows staining for E-cadherin (green fluorescence), DAPI (blue) and F-actin (phalloidin, red) in SSE cells. (A) and (C) show nontreated cells grown in 0.4 mmol/L Ca^+2^. In (B) and (D) the cells were treated for 48 h with 2.5 μmol/L CCT/0.4 mmol/L Ca^+2^. Cells treated with CCT lost their polygonal shape, E-cadherin redistributed from the cell membrane to the cytoplasm and F-actin elongations were prominent. Representative figures are from three different experiments.

The significant morphological changes induced by CCT suggested an effect on junctional proteins. To investigate the effect of CCT on cell–cell junction proteins and the cytoskeleton, cells were plated on glass coverslips precoated with fibronectin in 12-well plates until ∼60% confluent. Cells were then divided into groups and treated for 48 h as follows: (1) nontreated control; (2) nontreated in 0.4 mmol/L Ca^+2^; (3) CHX in control Ca^+2^ and; (4) CCT/0.4 mmol/L Ca^+2^. The cells were then fixed and stained for E-cadherin (green fluorescence) in combination with phalloidin (red) for F-actin filaments visualization. We chose E-cadherin because of its close association with Ca^+2^ signaling in a variety of cell types. As shown in Figure8, cells incubated in 0.4 mmol/L Ca^+2^ had normal cobblestone appearance with E-cadherin clearly delineating the cell membrane (Fig.8A and C). Treating the cells with CCT (in 0.4 mmol/L Ca^+2^) markedly altered the shape of the cells (Fig.8B) and translocated E-cadherin from the cell membrane to the cytoplasm (Fig.8B and D).

**Figure 9:**
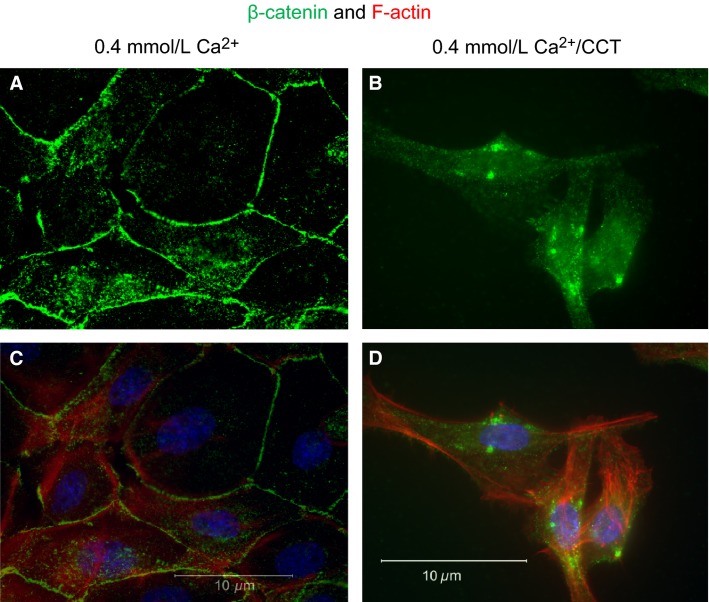
Effect of cinacalcet on *β*-catenin distribution. The figure shows staining for *β*-catenin (green fluorescence), DAPI (blue), and F-actin staining (phalloidin, red). (A) and (C) show nontreated cells grown in 0.4 mmol/L Ca^+2^. In (B) and (D), the cells were treated for 48 h with 2.5 μmol/L CCT/0.4 mmol/L Ca^+2^. Cells treated with CCT lost their polygonal shape, *β*-catenin redistributed from the cell membrane to the cytoplasm (green dots), and F-actin elongations were prominent. Representative figures are from three different experiments.

Staining for *β*-catenin, another junction protein, and phalloidin (Fig.9) showed similar response to CCT. Cells grown in 0.4 mmol/L Ca^+2^ had normal epithelial appearance, and *β*-catenin was clearly expressed at the cell membrane (Fig.9A and C). Treatment with CCT caused disruption of *β*-catenin, which also translocated from the cell membrane to the cytoplasm (area close to the nucleus, Fig.9B and D). Both Figures8D and 9D also show the disruption of F-actin and the remarkable formation of filopodia under CCT treatment.

**Figure 10:**
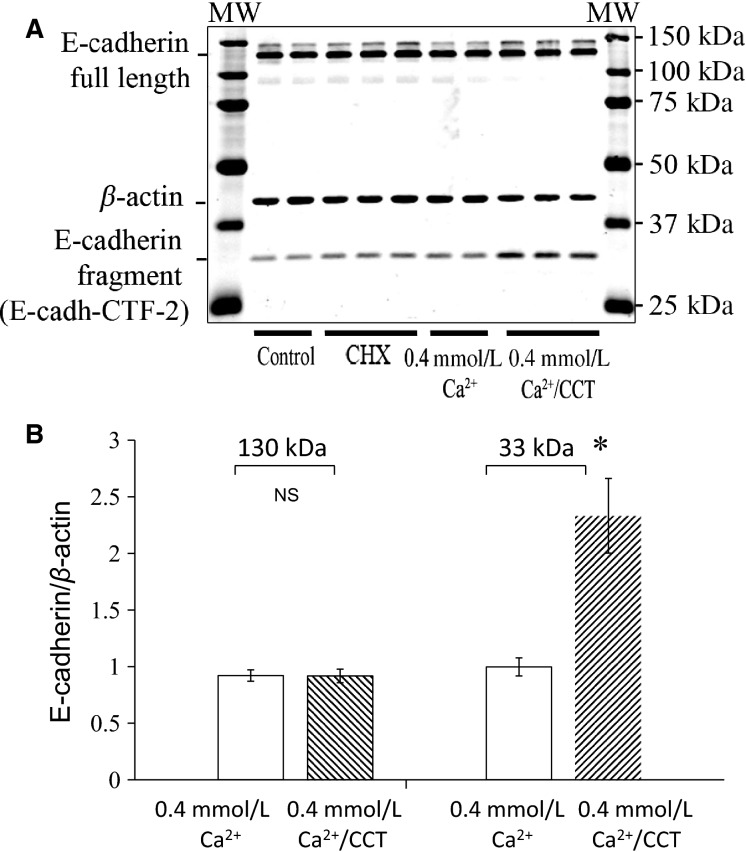
Effect of CaSR modulation on E-cadherin expression. (A), immunoblot analysis of lysates from control cells, cells treated with CHX, cells maintained in 0.4 mmol/L Ca^+2^ and cells treated with 2.5 μmol/L CCT/0.4 mmol/L Ca^+2^ for 48 h. The blot was stained for E-cadherin. Expression of E-cadherin fragment at 33 kD (E-cadh-CFT2) was significantly increased in CCT-treated cells. (B) is a bar graph showing that E-cadherin expression at the expected MW of 130 kD was not statistically different among the treatment groups. There was relative increase in expression of E-cadh-CFT2 in cells treated with CCT/0.4 mmol/L Ca^+2^ (2.3 folds, *P* < 0.01) as compared to nontreated cells. Data are from four different experiments. For each experiment, data from duplicates or triplicates were averaged for each sample to yield one data point (*N* = 4, *P < 0.02).

To further investigate these pronounced changes in localization of cell–cell junction proteins, we performed Western analysis and immunoblotting experiments, and determined the expression of E-cadherin, p120 and *β* catenin all of which are involved in maintaining the integrity of the adherens junctional complexes. E-cadherin is central to maintaining cell–cell adhesion; p120 and *β* catenin regulate E-cadherin by binding to its cytoplasmic tail (Reynolds and Roczniak-Ferguson 2004). For western blot analysis, cells were plated and grown in 25 cm^2^ flasks and then treated with CCT or CHX as described above. The cells were lysed and SDS-page and immunoblotting analysis were performed. As shown in Figure10, in cells treated with CCT, the expression of E-cadherin at the expected MW of 130 kD (full length) was not statistically different from control or from cells grown 0.4 mmol/L Ca^+2^ (Fig.10A and B). However, there was a significant increase (2.3-fold) in expression of a fragment of E-cadherin at the MW of 33 kD in CCT-treated cells (Fig.10A and B). This fragment is known as Ecad/CFT2 (David and Rajasekaran 2012) and is reported to result from proteolytic cleavage of E-cadherin caused by Ca^+2^ influx into the cell (Ito et al. 1999). Cells treated with CHX or 0.4 mmol/L Ca^+2^ did not show any significant change in full-length E-cadherin or in the 33 kD fragment (Fig.10A).

**Figure 11:**
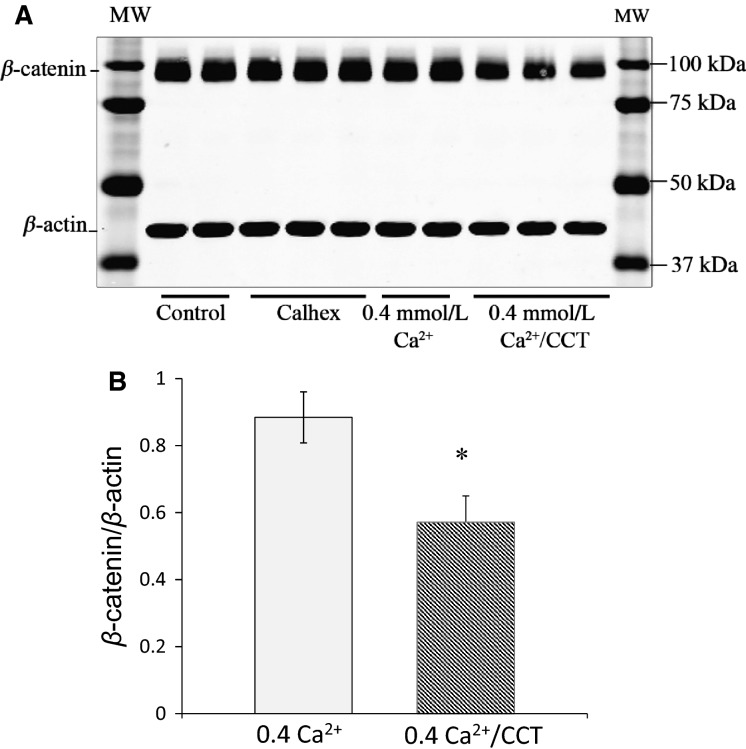
Effect of cinacalcet on *β*-catenin expression. (A), immunoblot analysis of lysates from control cells, cells treated with CHX, cells maintained in 0.4 mmol/L Ca^+2^ and cells treated with CCT/0.4 mmol/L Ca^+2^ for 48 h. The blot was stained for *β*-catenin. *β*-catenin expression at the expected MW of 100 kD was significantly decreased (by 35%) in CCT-treated cells. (B) is a bar graph showing relative expression of *β*-catenin in protein extracts from cells in 0.4 mmol/L Ca^+2^ and cells treated with CCT/0.4 mmol/L Ca^+2^ for 48 h. Data are from five different experiments. For each experiment, data from duplicates or triplicates were averaged for each sample to yield one data point (*N* = 5, * *P* < 0.02).

We next examined the effect of CCT treatment on *β*-catenin, a protein closely associated with E-cadherin in the cadherin–catenin junctional complex. As shown in Figure11, in cells treated with CCT, the expression of *β*-catenin (normalized to *β*-actin) was reduced by ∼35% (*P* < 0.02) (Fig.11B). There was no significant change in *β*-catenin in cells treated with CHX (Fig.11A).

**Figure 12:**
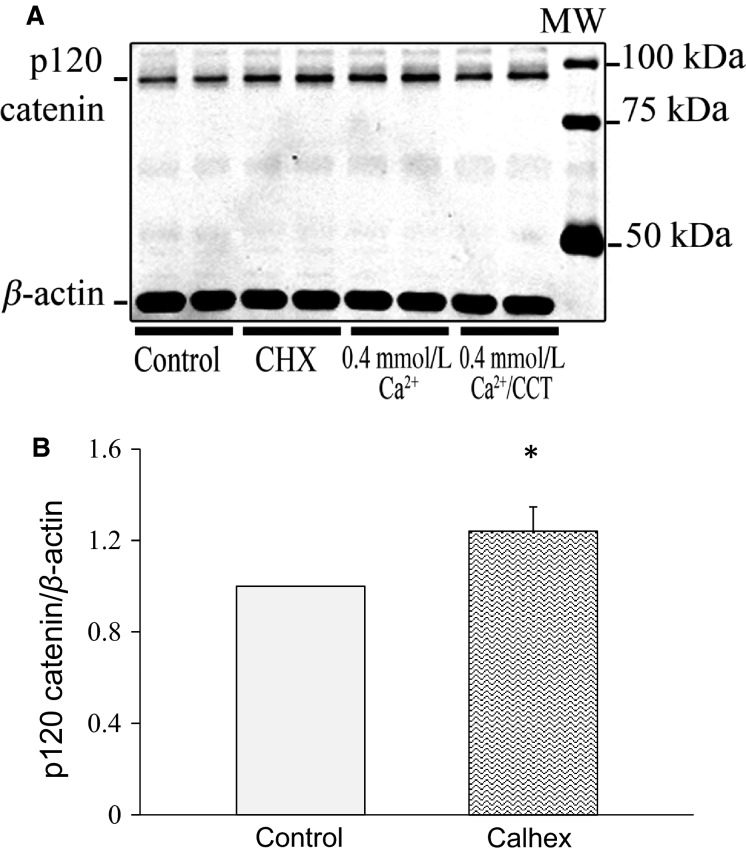
Effect of calhex on p120 catenin expression. (A), immunoblot analysis of lysates from control cells and cells treated with CHX. The blot was stained for p120 catenin. p120 catenin expression at the expected MW of 94 kD was higher (by 24%) in cells treated with CHX for 48 h. (B) is a bar graph showing relative expression of p120 catenin in control cells and cells treated with CHX. Data are from 6 different experiments. For each experiment, data from duplicates or triplicates were averaged for each sample to yield one data point (*N* = 6, *P < 0.05).

On the other hand, the expression of p120 catenin (expected MW 90–120 kD), a protein closely associated with the cadherin–catenin complex, was affected differently. Although the treatment with CCT did not cause a significant change in p120 catenin, we observed that in cells treated with CHX, p120 catenin was upregulated by ∼24% (*P* < 0.05) (Fig.12A and B).

**Figure 13:**
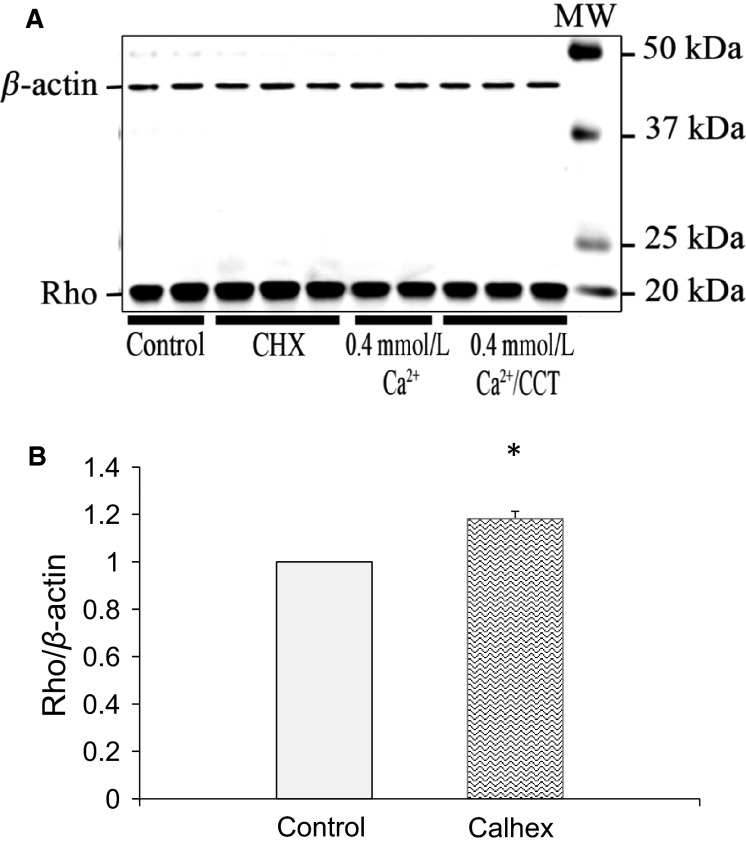
Rho expression in response to allosteric modulation of CaSR. (A), immunoblot analysis of lysates from control cells, cells treated with CHX, nontreated cells maintained in 0.4 mmol/L Ca^+2^ and cells treated with CCT/0.4 mmol/L Ca^+2^ for 48 h. The blot was stained for Rho. Rho expression at the expected MW of 20 kD was higher (by 18%) in cells treated with CHX. (B) is a bar graph showing relative increase in expression of Rho in cells treated with CHX as compared to nontreated control cells. Data are from three different experiments. For each experiment, data from duplicates or triplicates were averaged for each sample to yield one data point. (*N* = 3, *P < 0.03).

Because CCT induced a significant change in the morphology of the cells, we examined the effect of CCT on Rho. Rho proteins through their interactions with the junctional proteins and the cytoskeleton are reported to play an important role in the regulation of the shape, polarity, and motility of epithelial cells. To investigate the effect of CaSR modulation on Rho, we treated the cells with CCT or CHX for 48 h. The cells treated with CCT did not show a significant change in Rho, however, the cells exposed to CHX showed 18% (*P* < 0.03) increase in Rho expression (Fig.13A and B).

These experiments clearly demonstrated that modulating CaSR affected expression and/or localization of adherens junction proteins in esophageal cells.

## Discussion

### CaSR expression in the esophagus

Our study demonstrated that CaSR is expressed in the esophagus of normal human subjects as well as in esophageal tissues from patients suffering from different pathological conditions of the esophagus like eosinophilic esophagitis, Barrett’s esophagus, adenocarcinoma, and squamous cell carcinoma. In other tissues of the GI tract, CaSR has been linked to a number of functions including colonic fluid movement, fluid secretion, proliferation, and differentiation (for reviews see [Alfadda et al. 2014; MacLeod 2013a]). In an esophageal cell line HET1A, stimulating CaSR by spermine or Mg^2+^ resulted in intracellular calcium mobilization, activation of ERK1 and 2, and the secretion of cytokine IL-8 (Justinich et al. 2008; Mulder et al. 2009). In several tissues, the level of CaSR expression seems to vary among different pathological states. For example, CaSR expression is downregulated in colon cancer (Saidak et al. 2009). Although prominently expressed in the esophagus, the role of CaSR in health and disease is not yet clear. This study was conducted to further investigate the role of CaSR in the esophagus.

To do this, we developed a primary culture of esophageal squamous cells from the pig. The pig stratified squamous esophageal tissue and submucosal glands also express CaSR (Fig.2). The pig esophagus shares functional and morphological similarities with the human, including the presence of submucosal glands, and therefore is an excellent model of the human esophagus. Our experiments demonstrated that the squamous cells in culture showed robust proliferation, positive staining to esophageal epithelial markers CK13 and CK14 (Moll et al. 1982), and expressed CaSR. We used this model to investigate novel functions of CaSR particularly those that may affect the esophageal barrier function.

### Long-term stimulation of CaSR

The major finding of this study was that modulating the activity of CaSR in the esophageal epithelial cells had a significant effect on junctional proteins that play an important role in maintaining structural integrity of the epithelium. Long-term treatment (24–72 h) of the cultured squamous esophageal cells with the calcimimetic CCT in low Ca^+2^ media (0.4 mmol/L), decreased cell proliferation, caused a change in cell shape (Fig.4D and E), and induced ruffling and formation of filopodia (Figs.8D, 9D). A decrease in cell proliferation was observed similarly in renal cells (Chen et al. 2011) and an effect of calcimimetics on cell morphology has been reported in HEK cells expressing exogenous CaSR (Bouschet et al. 2007). In our experiments, the CCT-induced change in cell shape was remarkable. The loss of cobblestone appearance to spindle-shape and the internalization of E-cadherin and *β*-catenin are hallmarks of epithelial to mesenchymal transition (EMT) (Zeisberg and Neilson 2009), and have been attributed to changes in activation of Rho proteins (reported to regulate tight junctions) (Davies et al. 2006) or ARF6 (Bouschet et al. 2007).

In our experiments, treatment with CCT-induced ruffling and formation of filopodia. To the best of our knowledge, this is the first report about ruffling or filopodia formation in epithelial cells expressing endogenous CaSR. The presence of F-actin filaments was demonstrated by phalloidin staining, which was prominent in protrusions of CCT-treated cells (Figs.8D, 9D). In several cell types, the formation of filopodia indicates reorganization of the actin cytoskeleton. This mechanism plays an important role in cell migration, regulation of cell shape, and cell–cell contact formation (Vasioukhin et al. 2000; Mattila and Lappalainen 2008). Our data indicate that CaSR activation plays a role in mediating this effect.

The effect of calcimimetics on cell morphology, filopodia formation, and proliferation strongly point to a role of CaSR in affecting tight junction proteins. In support of this observation, we showed that treatment with CCT caused translocation of E-cadherin and *β*-catenin from the cell membrane to the cytoplasm (Figs.8B, 9B). The loss of E-cadherin from the cell membrane was accompanied by a decrease in *β*-catenin expression in the cells. Strikingly, the extension of filopodia in CCT-treated cells was accompanied by disruption and internalization of E-cadherin and *β*-catenin confirming that sustained activation of the CaSR with CCT disrupts cell–cell contact formation.

We further confirmed the disruption of E-cadherin in CCT-treated cells by Western analysis and showed that expression of a proteolytic fragment of E-cadherin at 33 kD was markedly increased. In other epithelial cells, this fragment of E-cadherin has been reported to result from proteolysis of extracellular and cytoplasmic domains of E-cadherin induced by calcium influx into the cell (Ito et al. 1999) or from activation of Cdc42 and matrix metalloproteinase (MMP) (Grieve and Rabouille 2014). The proteolytic cleavage of E-cadherin and generation of 33 kD fragment has been studied in a number of epithelial cells (Baki et al. 2001; Marambaud et al. 2002) and was reported in the esophageal epithelium of reflux esophagitis patients (Jovov et al. 2011). These proteolytic fragments may play an important role as oncogenes (David and Rajasekaran 2012) or as signaling molecules (Ferber et al. 2008). It is possible that one or more proteolytic step (s) of E-cadherin was activated by CCT treatment. In our study, the loss of *β*-catenin from the cell junctions in CCT-treated cells further confirmed the degradation of cell–cell contacts (Ito et al. 1999). Moreover, reduced *β*-catenin expression in CCT-treated cells is a strong indication that the E-cadherin/*β*-catenin complex has been destabilized. The dissolution of intercellular junctions and acquisition of filopodia is reminiscent of “epithelial cell scattering” that gives the epithelial cells a motile phenotype (Palacios and D’Souza-Schorey 2003).

### Effect of CCT on CaSR expression and localization

Our experiments indicated that long-term treatment of the cells with CCT caused redistribution of CaSR from the cell membrane to the endoplasmic reticulum–nuclear area (Fig.6B). On the other hand, our immunoblotting experiments did not show a significant change in CaSR protein expression at the expected MW of 130 kD following CCT treatment, indicating that CCT affected the distribution and trafficking of CaSR between the cell membrane and the endoplasmic reticulum rather than the biosynthesis of the receptor.

We treated the cells with CCT in the presence of 0.4 mmol/L Ca^+2^ in the media. CCT is an allosteric modulator of CaSR that increases the receptor’s sensitivity to Ca^+2^ by shifting the response curve to the left and has been reported to induce its effect at a lower concentration of Ca^+2^ (Nemeth et al. 1998). At full concentration of Ca^+2^ of 1.2 mmol/L, the receptor would already be occupied by its primary agonist and therefore the effect of CCT might be masked. It is important to note that in our experiments, cells grown in 0.4 mmol/L Ca^+2^ had normal proliferation and morphology (Figs.8A, 9A).

Because we were concerned that CCT treatment might cause apoptosis of the esophageal cells as it was described in other cell types (Li et al. 2009), we confirmed that although the total number of cells was reduced after treatment, the relative number of live cells at the same time frame of 24 or 48 h was not significantly decreased by CCT treatment.

### The effects of negative modulation of CaSR

Calhex 231 (CHX) is a potent negative allosteric modulator of CaSR that inhibits inositol phosphate increases caused by Ca^+2^ activation of the receptor (Petrel et al. 2003). We showed here that incubation of the cells with CHX unexpectedly enhanced cell proliferation (Fig.5A) and did not have an effect on protein expression of E-cadherin and *β*-catenin over the course of 48 h.

Cells treated with CHX also showed upregulation of p120 catenin (Fig.2), an adherens junction protein closely associated with E-cadherin and *β*-catenin and with the regulation of Rho activity. Rho-GTPases like Rho, Rac, and Cdc42 are a family of small guanosine triphosphatases that play an important role in actin remodeling in a variety of cells (Etienne-Manneville and Hall 2002). Rho proteins are activated by binding to GTP, and they regulate multiple cell functions like tight junction formation and motility (Jaffe and Hall 2005). Several reports in the literature link expression of p120 catenin and Rho activity (Perez-Moreno et al. 2006; Wildenberg et al. 2006; Epifano et al. 2014). p120 catenin has been reported to interact directly with GDP-bound RhoA to inhibit Rho activity (Anastasiadis et al. 2000). Our data indicated an increase in total Rho expression in CHX-treated cells (Fig.3) alongside increased expression of p120 catenin (Fig.2). The interaction of these two proteins regulates E-cadherin recycling and the junctional complexes (Fukata and Kaibuchi 2001). It is conceivable that CHX-induced increase in p120 catenin stabilizes Rho and contributes to maintenance of adherens junctions.

In conclusion, we have confirmed the presence of CaSR in normal and diseased human esophagus. We established a cell culture model of esophageal squamous cells and showed that sustained CaSR activation by CCT caused the cells to lose their epithelial phenotype and their adherens junction complexes, and to acquire extensive filopodia. We demonstrated that the modifications of the actin cytoskeleton involved cleavage of E-cadherin and a decrease in *β*-catenin expression. On the other hand, treating the cells with CHX, a negative allosteric modulator of CaSR, increased the expression of p120 catenin and the small protein GTPase Rho. This study demonstrates that modulation of CaSR in esophageal cells alters distribution and expression of adherens junction proteins and could therefore play an important role in the homeostasis and barrier function of the epithelium.
